# Diagnostic analysis of a new sign (superior sagittal sinus distention) on spontaneous intracranial hypotension MR imaging: a retrospective study

**DOI:** 10.1186/s12880-025-01975-9

**Published:** 2025-10-24

**Authors:** Weiling Cheng, Weili Xie, Rixin Zhang, Junyuan Zhong, Yongmei Hu, Jiali Guo, Guangbin Xie

**Affiliations:** 1 Department of Neurosurgery, The First Affiliated Hospital, Jiangxi Medical College, Nanchang University, Nanchang, Jiangxi China; 2 Department of Radiology, The First Affiliated Hospital, Jiangxi Medical College, Nanchang University, Nanchang, Jiangxi China; 3 Clinical Research Center For Medical Imaging, Nanchang, Jiangxi China; 4 Department of Radiology, Ganzhou People’s Hospital, Ganzhou, Jiangxi China; 5 Department of Radiology, Xinyu People’s Hospital, Xinyu, Jiangxi China; 6 Department of Neurology, The First Affiliated Hospital, Jiangxi Medical College, Nanchang University, Nanchang, Jiangxi China

**Keywords:** Superior sagittal sinus distention, Spontaneous intracranial hypotension, Magnetic resonance imaging, T2-weighted image, Diagnostic analysis

## Abstract

**Objectives:**

Venous distention may occur in the early stages of spontaneous intracranial hypotension (SIH) as a compensatory mechanism. We hypothesized that analysis of superior sagittal sinus distention (SSSD) on T2-weighted axial images, in combination with other imaging findings, could enhance diagnostic accuracy for SIH.

**Methods:**

This retrospective study analyzed data from patients diagnosed with SIH across three institutions. Data from one institution served as the training set, while data from the remaining two institutions constituted the validation set. Patients with non-spontaneous hypotensive headaches from the same institution during the study period were selected as controls. Two senior neuroradiologists and one junior neuroradiologist evaluated SSSD along with four other imaging findings.

**Results:**

In the training set, senior neuroradiologists demonstrated 84.48% sensitivity in identifying SSSD, significantly higher than other findings, with 89.66% specificity, slightly lower than alternative findings. In the validation set, both sensitivity and specificity for SSSD reached 100%, indicating high diagnostic accuracy. When assessing differences in interpretation among radiologists of varying experience, the junior neuroradiologist showed lower sensitivity (67.24%) for SSSD compared to pachymeningeal thickening. However, their sensitivity improved substantially in the validation set relative to other findings, reaching 60%.

**Conclusion:**

SSSD demonstrates higher sensitivity compared to other imaging findings and exhibits consistent reliability across different institutions and varying levels of neuroradiologist expertise. When integrated with additional imaging findings, it can substantially improve SIH diagnosis.

**Supplementary Information:**

The online version contains supplementary material available at 10.1186/s12880-025-01975-9.

## Introduction

Spontaneous intracranial hypotension (SIH) is a disorder caused by cerebrospinal fluid (CSF) leakage, typically resulting from dural tears, meningeal diverticula leakage, or CSF-venous fistulas [1]. The primary clinical presentation is orthostatic headache, often accompanied by additional complex symptoms [2]. Consequently, patients may be initially misdiagnosed with alternative conditions, such as pituitary tumors or Chiari I malformations, leading to diagnostic delays and inappropriate treatment [3].

The characteristic imaging findings of SIH include pachymeningeal thickening, venous sinus distention, subdural effusion, pituitary enlargement, and brain sagging [4], with pachymeningeal thickening being most frequently observed. However, approximately 25% of patients may not exhibit this feature in early stages [5]. Furthermore, assessment of pachymeningeal thickening requires cranial MR enhancement sequences. While studies suggest T2 fluid-attenuated inversion recovery (FLAIR) sequences may partially substitute for contrast-enhanced MRI in evaluating dural thickening [6, 7], this approach increases interpretative demands on radiologists. Thus, relying solely on early pachymeningeal thickening as a diagnostic indicator for SIH has inherent limitations.

Research by Chen et al. demonstrated that venous distention was more prevalent (> 75%) and manifested earlier on brain MRI in SIH patients [8]. Previous studies primarily identified venous distention by examining the transverse and straight sinuses [9, 10]; however, accurate assessment of transverse sinus contour can be challenging due to significant signal variations. Additionally, straight sinus visibility may be compromised by scan slice thickness. T2-weighted axial images enable continuous observation of superior sagittal sinus morphological alterations at multiple levels with distinct edge contours. Therefore, this study aims to facilitate SIH diagnosis by analyzing superior sagittal sinus distention (SSSD) on T2-weighted axial images in combination with other imaging findings.

## Materials and methods

### Participants

**Fig. 1:**
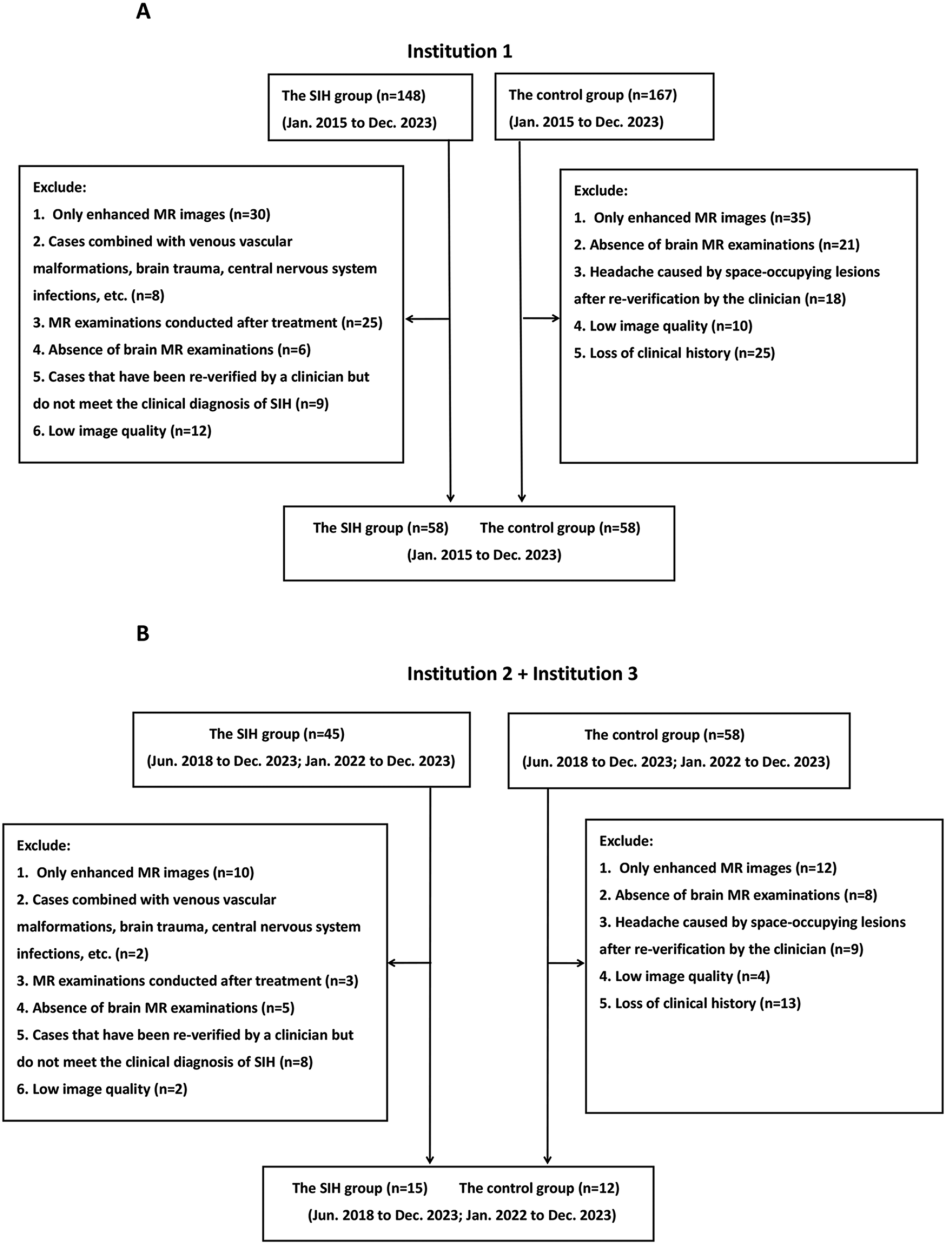
Flow chart showing participants inclusion and exclusion

The study included patients diagnosed with SIH from three institutions: Institution 1 (January 2015 to December 2023), Institution 2 (June 2018 to December 2023), and Institution 3 (January 2022 to December 2023). Cases were identified using the keyword “intracranial hypotension” in the case management system, followed by clinical diagnosis verification by two neurologists according to established SIH diagnostic criteria [11, 12]. Inclusion criteria were: (1) confirmed clinical diagnosis of SIH; (2) complete brain MRI examinations; and (3) pre-treatment MR images. For patients who underwent both non-enhanced and contrast-enhanced brain MRI, only non-enhanced images were included in this study. The case selection process is illustrated in Fig. 1. The control group comprised age- and sex-matched patients diagnosed with other headache disorders, including migraine, tension-type headache, trigeminal autonomic headache, and headaches of unknown origin, recruited during the same study period. All control participants underwent rigorous screening to exclude SIH. Given the retrospective design, controls were required to have ≥ 6 months of clinical follow-up demonstrating diagnostic stability without subsequent development of SIH symptoms or suggestive imaging findings. In this retrospective study, standardized case report forms were utilized to systematically collect baseline clinical data from all enrolled participants. The collected parameters included: (1) demographic characteristics (age and sex); (2) headache duration, operationally defined as the interval (in days) between symptom onset and initial MR imaging; and (3) associated neurological symptoms (including nausea and vomiting), which were documented as dichotomous variables (present/absent).

### Ethical approval

All procedures performed in the studies inyolving human participants were in accordance with the ethical standards of the institutional and/or national research committee and with the 1964 Helsinki Declaration and its later amendments or comparable ethical standards. This retrospective study has been approved by the Medical Ethics Committee (approval number IIT2024186-1), with informed consent waived for all participants.

### Magnetic resonance imaging examination

In this study, all pre-treatment patients underwent scanning using a 1.5T or 3.0T MRI system (including Amira, Siemens; Avanto, Siemens; TrioATim, Siemens; Skyra, Siemens; Opitima360, GE Healthcare; SIGNA Pioneer, GE Healthcare). The imaging protocol included: (1) T1-weighted sagittal plane images (TR 200 ms, TE 4.76 ms, FA 90; TR 194 ms, TE 4.76 ms, FA 70; TR 250 ms, TE 2.46 ms, FA 70; TR 240 ms, TE 2.46 ms, FA 70; TR 2250 ms, TE 8.7 ms, FA 160; TR 2236 ms, TE 7.3 ms, FA 111); (2) T2 weighted axial images (TR 4500 ms, TE 106 ms, FA 120; TR 3500 ms, TE 100 ms, FA 150; TR 4000 ms, TE 113 ms, FA 140; TR 3000 ms, TE 117 ms, FA 90; TR 4781 ms, TE 121 ms, FA 160; TR 5584 ms, TE 123 ms, FA 110); (3) T2-weighted FLAIR images (TR 8500 ms, TE 81 ms, FA 150; TR 8400 ms, TE 90 ms, FA 150; TR 8000 ms, TE 79 ms, FA 120; TR 8400 ms, TE 78 ms, FA 150; TR 8400 ms, TE 80 ms, FA 160; TR 8400 ms, TE 136 ms, FA 160). When T2 weighted axial scanning was carried out, all images were in accordance with a layer thickness of 5 mm, layer spacing of 1.5 mm, and a field of view (FOV) of 240 × 240 mm. All brain MRI scans were acquired to the anterior commissure-posterior commissure (AC-PC) reference plane. T1-weighted sagittal images were predominantly utilized for the evaluation of brain sagging and pituitary enlargement. T2-weighted axial images were primarily employed to assess SSSD, as well as to identify the presence of subdural effusion or hemorrhage. T2-FLAIR images were chiefly used to examine pachymeningeal thickening and to exclude other potential intracranial abnormalities, such as neoplastic or inflammatory lesions.

### Image analysis

**Fig. 2:**
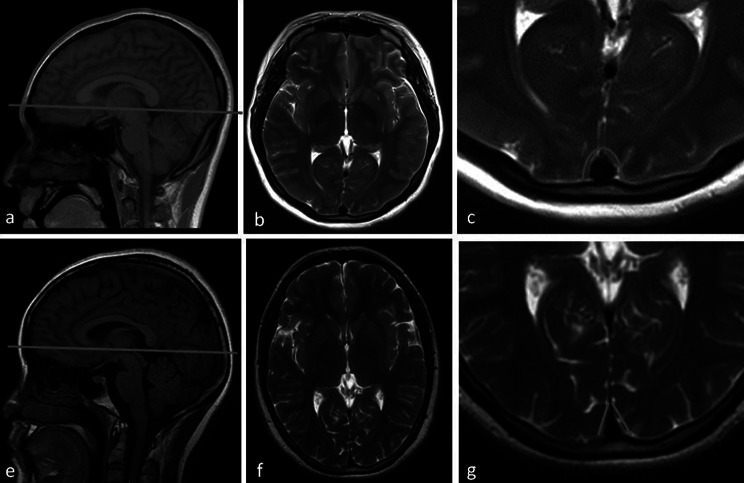
Superior sagittal sinus distention (**a-c**) and normal superior sagittal sinus image (**e-g**)

Data from Institution 1 constituted the training set, while data from Institutions 2 and 3 formed the validation set. Two senior neuroradiologists independently evaluated the training set data for SSSD and other imaging features, blinded to clinical information. As shown in Fig. 2, normal sagittal sinuses appear triangular on axial images with straight, well-defined edges. Distention was defined as edge curvature or noticeable fullness resulting in circular or oval morphology. Discrepancies between senior neuroradiologists were resolved by consensus. The same radiologists analyzed the validation set to assess the external validity of SSSD as a diagnostic indicator. Additionally, a junior neuroradiologist independently interpreted both datasets using identical methodology to evaluate interpretation variability across experience levels.

### Statistical analysis

Statistical analyses were performed using SPSS version 26.0. The Shapiro-Wilk test assessed normality of continuous variables. Normally distributed data were analyzed using independent sample t-tests. Categorical variables were compared using chi-square tests. Statistical significance was set at *P* < 0.05.

## Results

The demographic and clinical data of all patients are presented in Table 1. In terms of age distribution, the average age of patients in the SIH group in the training set was 41.17 ± 10.86 years, compared to 41.29 ± 10.77 years in the control group. In the validation set, the average age of the SIH group was 48.20 ± 16.68 years, whereas that of the control group was 40.67 ± 14.12 years. Gender composition analysis revealed that the proportion of female patients in the SIH group was 72.4% (42/58) in the training set and 68.9% (40/58) in the control group. In the validation set, the proportion of female patients in the SIH group was 80.0% (12/15), compared to 58.3% (7/12) in the control group. Statistical analysis demonstrated that, although the majority of SIH patients were young and middle-aged women, there were no statistically significant differences in age or gender distribution between the groups (*p* > 0.05). Symptom analysis of the training set indicated that the duration of headache was significantly longer in the SIH group than in the control group (16.16 ± 19.68 days vs. 8.66 ± 8.49 days, *p* = 0.009), and the incidence of vomiting was significantly higher (21 cases vs. 4 cases, *p* < 0.001). However, due to the limited sample size (*n* = 27) in the validation set, no statistically significant differences were observed in headache duration or vomiting incidence between the groups (*p* > 0.05). Based on the combined analysis of both datasets, it can be concluded that the incidence of nausea and vomiting is higher among patients with SIH.

**Table 1:** Participants demographics in training and validation sets

	The SIH group	The control group	*P* value
Training Set			
Age, mean (SD), y	41.17±10.86	41.29±10.77	0.952
Sex(male/female)	16/42	18/40	0.683
Symptom duration(day)	16.16±19.68	8.66±8.49	0.009*
nausea	18	11	0.133
vomit	21	4	0.000*
Validation Set			
Age, mean (SD), y	48.20±16.68	40.67±14.12	0.224
Sex(male/female)	3/12	5/7	0.221
Symptom duration(day)	11.73±16.34	11.33±7.76	0.939
nausea	5	3	0.637
vomit	5	1	0.537

*p < 0.05

Diagnostic performance of MRI features analysis by senior neuroradiologists (Tables 2 and 3) revealed SSSD had the highest sensitivity (84.48%), significantly exceeding other imaging features. Although its specificity (89.66%) was slightly lower than other features in the training set, both sensitivity and specificity reached 100% in the validation set, demonstrating excellent diagnostic accuracy.

**Table 2:** Diagnostic performance of SIH based on MRI features by senior neuroradiologists in training set

MR finding	SEN(%)	SPE(%)	ACC(%)	PPV(%)	NPV(%)
Superior sagittal sinus distention	84.48	89.66	87.07	89.09	85.25
Pachymeningeal thickening	58.62	100.00	79.31	100.00	33.33
Subdural effusion(hematoma)	15.52	100.00	57.76	100.00	54.21
Brain sagging	10.34	100.00	55.17	100.00	58.00
Pituitary enlargement	44.83	98.28	71.55	96.29	64.04

**Table 3:** Diagnostic performance of SIH based on MRI features by senior neuroradiologists in validation set

MR finding	SEN(%)	SPE(%)	ACC(%)	PPV(%)	NPV(%)
Superior sagittal sinus distention	100.00	100.00	100.00	100.00	0
Pachymeningeal thickening	20.00	100.00	55.55	100.00	50.00
Subdural effusion(hematoma)	0	100.00	44.44	0	55.55
Brain sagging	0	100.00	44.44	0	55.55
Pituitary enlargement	13.33	91.66	48.15	66.67	84.62

Junior radiologist interpretation (Tables 4 and 5) showed higher diagnostic performance for pachymeningeal thickening in the training set (sensitivity 72.41%, specificity 91.38%, accuracy 81.89%, positive predictive value (PPV) 89.36%, negative predictive value (NPV) 76.81%). While SSSD sensitivity was initially lower (67.24%), it showed marked improvement in the validation set compared to other features, reaching 60%.

**Table 4:** Diagnostic performance of SIH based on MRI features by junior neuroradiologist in training set

MR finding	SEN(%)	SPE(%)	ACC(%)	PPV(%)	NPV(%)
Superior sagittal sinus distention	67.24	89.66	78.45	86.67	73.24
Pachymeningeal thickening	72.41	91.38	81.89	89.36	76.81
Subdural effusion(hematoma)	10.34	96.55	53.45	75.00	51.85
Brain sagging	1.72	100.00	50.86	100.00	50.00
Pituitary enlargement	29.31	91.38	60.34	77.27	56.38

**Table 5:** Diagnostic performance of SIH based on MRI features by junior neuroradiologists in validation set

MR finding	SEN(%)	SPE(%)	ACC(%)	PPV(%)	NPV(%)
Superior sagittal sinus distention	60.00	91.66	74.07	90.00	64.71
Pachymeningeal thickening	20.00	100.00	55.56	100.00	50.00
Subdural effusion(hematoma)	6.66	100.00	48.15	100.00	46.15
Brain sagging	0	100.00	44.44	0	44.44
Pituitary enlargement	13.33	91.67	48.15	66.66	45.83

## Discussion

This multi-institutional study of 73 SIH patients demonstrated that SSSD exhibits higher sensitivity compared to traditional imaging features. This finding showed remarkable consistency across institutions and reader experience levels, complementing the relatively lower sensitivity of other imaging features and enhancing diagnostic accuracy through comprehensive analysis.

The findings of this study suggest that there are some differences between senior and junior neuroradiologists in their ability to recognize SSSD image features. These differences may be attributed to the impact of accumulated clinical experience on diagnostic proficiency. Senior radiologists demonstrate greater diagnostic confidence when identifying SSSD signs. Importantly, the SSSD signs introduced in this study exhibit higher sensitivity compared to existing imaging signs. Furthermore, junior radiologists are able to acquire proficiency in recognizing these signs after brief instruction. This indicates that through systematic, standardized training and targeted education, the efficiency with which radiologists identify SSSD signs associated with low intracranial pressure headache can be significantly enhanced, ultimately improving the imaging diagnostic pathway for this condition.

The Monro-Kellie hypothesis states that the combined volume of brain tissue, CSF, and intracranial blood remains constant, with changes in one component leading to compensatory changes in others [13]. Recent research has shown that brain tissue volume is not static; decreased CSF correlates with reduced brain tissue volume [14]. Consequently, CSF loss initially triggers compensatory increases in intracranial blood volume, manifesting as intracranial congestion. Given the greater compliance of veins compared to arteries [15], this congestion predominantly affects the venous system.

The venous sinuses serve as the primary intracranial venous drainage system [16], maximally compensating for CSF volume loss. Upon CSF leak cessation, venous sinus congestion is typically the first imaging finding to normalize [9]. Other imaging features associated with CSF leakage, such as pachymeningeal enhancement and subdural effusion, develop later. When venous sinus distention reaches its elastic limit, structural brain changes may occur, including brain sagging [17].

In our cohort, mean headache duration was 16.16 ± 19.68 days in the training set and 11.73 ± 16.34 days in the validation set, indicating early-stage disease. This timing explains the higher prevalence of venous sinus distention compared to other features, while subdural effusion and brain sagging were less common, consistent with previous findings [8].

The higher sensitivity of pachymeningeal thickening reported by the junior radiologist in the training set likely reflects potential overinterpretation. Pachymeningeal thickening typically manifests as diffuse, smooth dural thickening on contrast-enhanced T1-weighted sequences [18]. Interpretation of this feature on T2 FLAIR sequences presents additional challenges for less experienced readers. Moreover, pachymeningeal thickening correlates with disease duration in SIH [5], typically developing after SSSD as a consequence of compensatory dural venous congestion following venous sinus insufficiency.

Image acquisition parameters significantly influence feature interpretation. In the training cohort, all scans were performed at a single institution with strict adherence to the AC-PC line alignment for axial T2-weighted imaging, which provided optimal visualization of SSSD signs. However, in the validation cohorts (from two external institutions), some cases could only be evaluated on T2-weighted images acquired at slightly offset planes (above/below the AC-PC line) due to variations in local protocols. This introduced challenges in consistent SSSD detection across all cases. Based on these findings, we strongly advocate for standardizing AC-PC line alignment as the baseline for brain MRI acquisition, as it represents the current gold standard in neuroimaging, ensures reproducible evaluation of subtle findings like SSSD, and facilitates reliable multicenter comparisons [19].

Current protocols lack standardized scanning baselines for transverse sinus evaluation in coronal views. Normal anatomic variations in transverse sinus development [20], including unilateral hypoplasia with contralateral compensation, can confound interpretation of pathologic distention. While developmental variations also occur in the superior sagittal sinus—primarily anterior third hypoplasia—our analysis focused on the posterior two-thirds, providing more reliable assessment compared to transverse sinus evaluation [21].

This investigation has several limitations. Despite the multi-institutional design, the low incidence of SIH resulted in a relatively small sample size, with only two centers contributing cases from the past five years. Further validation in larger cohorts is warranted. Additionally, SSSD shows temporal dependence; patients with prolonged symptoms may not demonstrate this feature prominently. Therefore, it should not serve as an independent diagnostic criterion but rather be integrated with other imaging features. This finding holds particular value for patients with contraindications to contrast administration. Finally, our study did not directly compare SSSD with other venous sinus enlargement patterns, partly due to interpretative challenges with certain distention phenomena.

## Conclusion

This study introduces SSSD as a novel imaging sign that enhances early diagnosis of SIH. This approach provides accurate and reliable diagnostic information without contrast administration, offering particular value for patients with contrast allergies or financial constraints.

## Supplementary Information

Below is the link to the electronic supplementary material.

Supplementary Material 1

## Data Availability

Data is provided within the supplementary information file.

## Abbreviations

AC-PC: Anterior commissure-posterior commissure
CSF: Cerebrospinal fluid
FLAIR: Fluid-attenuated inversion recovery
FOV: Field of view
NPV: Negative predictive value
PPV: Positive predictive value
SIH: Spontaneous intracranial hypotension
SSSD: Superior sagittal sinus distention
